# A STUDY OF ANDROGEN AND ESTROGEN RECEPTORS α, β IN SKIN TAGS

**DOI:** 10.4103/0019-5154.60345

**Published:** 2010

**Authors:** Omar El Safoury, Lila Rashid, Magdy Ibrahim

**Affiliations:** *From the Department of Dermatology, Faculty of Medicine, Cairo University, Egypt*; 1 *From the Department of Biochemistry, Faculty of Medicine, Cairo University, Egypt*; 2 *From the Department of Obstetrics and Gynecology, Research Biostatistics Unit, Management Team, EBM Unit, MEDC, Faculty of Medicine, Cairo University, Egypt*

**Keywords:** *Androgen receptors*, *acanthosis nigricans*, *body mass index*, *estrogen α receptors*, *estrogen β receptors*, *sex hormone receptors*, *mast cell*, *skin tags*, *obesity*

## Abstract

**Background:**

In women, the age of 50 is suggested to be the turning point of life at which the development of skin tags comes to a stop. A major event that occurs around this period of life is menopause/andropause. After menopause, estrogen receptors amounts decrease significantly. As skin is considered as the largest nonreproductive target on which estrogens and androgens act, we assume a possible relationship between the pathogenesis of skin tags and sex steroid balance. Another phenomenon is the association of skin tags in obese patients, which may also be explained by the interplay of sex steroids and their receptors in skin tags.

**Aims:**

Here we see that in obese patients, hyperandrogenism occurs as a result of hyperinsulinemia as well as peripheral conversion of estrogens into androgens in the excessive adipose tissue. To examine the possible role of androgen and estrogen receptors in etiopathogenesis of skin tags.

**Materials and Methods:**

To examine these hypotheses, we measured the level of androgen and estrogen receptors (both α and β) in skin tags compared to control. We also correlated the level of receptors to body mass index, and compared those levels in patients with acanthosis nigricans compared to normal.

**Results:**

The level of estrogen receptors (both α and β) was significantly higher in skin tags than in controls with a *P* value of 0.004 and 0.001, respectively. The same upsurge was found for androgen receptors in skin tags relative to control with a *P* value of 0.001. No statistically significant difference in receptor level was found either among patients with acanthosis nigricans and those without, or in correlation to body mass index (our participants were overweight non diabetic).

**Conclusion:**

These results suggest the possible role of androgen and estrogen receptors in etiogenesis of skin tags, and propose that the neck is an androgen dependent area just similar to the axillae and the groins, though hairless.

## Introduction

Estrogen, the predominant steroid responsible for secondary sexual characteristics in females, influences the function of all major organ systems within the body. Skin constitutes the largest nonreproductive target on which estrogen acts.[1] Estrogens are C-18 steroids synthesized from cholesterol in the ovary and in peripheral tissues, especially adipose tissue. In postmenopausal women, estrogen synthesis is restricted to peripheral tissues. In the latter, the enzyme aromatase is responsible for conversion of the androgen androstenedione to estradiol. While aromatase enzyme is present in the ovarian theca cells, bone, brain, and skin, the majority of peripheral production of estrogen occurs in the skin.[1]

Several phenomena point to a tight relationship between skin homeostasis and sex steroids. Among those are skin tags that manifest exclusively in postpubertal life,[2] and stop developing following menopause,[2] which can be theoretically explained by their dependence on sex steroids, the receptors of which fade following menopause.[3] This is in addition to other phenomena such as increased incidence of soft fibromas during pregnancy.[4]

On the other hand, among massively obese males, who exhibit decline in total and free testosterone as well as elevated esrtadiol levels,[5] and among obese females with syndrome X who suffer high androgen levels, decreased sex hormone binding globulin level and polycystic ovaries are observed.[6] These phenomena further support the theory that estrogens and androgens are involved in the pathogenesis of various cutaneous disorders among which are skin tags.

The aim of this study is to examine the possible role of androgen and estrogen receptors in etiogenesis of skin tags.

## Materials and Methods

This study included 15 participants, including 12 females and 3 males, attending the outpatient clinic of the dermatology unit. Their complaint was cosmetically unappealing skin tags. Mean age was 34.20 ± 6.603 years. All participants were nondiabetic. Patients were examined for the number of skin tags, color, the presence, or absence of acanthosis nigricans, body mass index (BMI), in addition to the routine general examination and examination of the skin for concomitant pathology.

BMI was calculated according to the following equation:[7]

BMI = weight (Kg)/height (m^2^)

The levels of androgen receptors (AR), estrogen receptors α (ERα), and estrogen receptors β (ERβ) were estimated by taking a skin snip spanning one of the skin tags as a study sample, and a piece of the surrounding area as a control sample and evaluating each separately by reverse transcription-polymerase chain reaction (RT-PCR).

## Reverse transcription-polymerase chain reaction

### RNA extraction

Total RNA was extracted from the study sample and control sample by the acid guanidinum thiocyanate-phenol-chloroform method R1.[8] RNA content and purity were measured by a UV spectrophotometer. The A260/A280 ratio was 1.8 to 2.0. RNA was of high integrality, being detected by agarose gel electrophoresis.

#### Reverse transcription-polymerase chain reaction procedure

RT-PCR was performed using the extracted RNA for the detection of estrogen and androgen receptor genes. For the amplification of the targets genes, reverse transcription and PCR were run in two separate steps. Equal amounts of total RNA (6 μg) were heat denatured and reverse transcribed by incubation at 42°C for 90 min with 12.5 U avian myeloblastosis virus (AMV) reverse transcriptase (Promega Corp., Madison, WI), 20 U ribonuclease inhibitor RNasin (Promega Corp.), 200 nM deoxy-nucleoside 5'-triphosphate mixture, and 1 nM oligo-dT primer in a total volume of 30 μl of 1x avian myeloblastosis virus reverse transcriptase buffer. The reactions were terminated by heating at 97°C for 5min and cooling on ice. cDNA samples were amplified in 50 μl of ×1 PCR buffer in the presence of 2.5 U Taq DNA polymerase (Promega Corp.), 200 nM deoxy-nucleoside 5'-triphosphate mixture, and the appropriate primer pairs (1 nM of each primer). These sets of primers, their annealing temperatures, and product sizes are listed in Table 1.

**Table 1:** The oligonucleotide primers sequence of studied genes

	Primer sequence	Annealing temperature	Product size
Estrogen	Forward primer: 5'CGGAGCACGGGGACGGGTATC-3'	65°C	541 bp
receptor α	Reverse primer: 5'-AAGACGAAGGGGAAGACGCACATC-3'.		
Estrogen	Forward primer: 5'TCTGGCATCCTCTTGTTGCT-3'	55°C	418 bp
receptor β	Reverse primer: 5'- CACAGCCAGCACTATAGGTCTTT-3'.		
Androgen	Forward primer: 5'TACCATGCTGTTTGGTTCA-3'.	65°C	208 bp
receptor	Reverse primer: 5'-TCAAGCTACCAATGACTTTC-3'.		

PCR started with an initial denaturing cycle at 97°C for 5min, followed by a variable number of amplification cycles comprising denaturation at 96°C for 1.5 min, annealing for 1.5min, and extension at 72°C for 3 min. A final extension cycle of 72°C for 15 min was performed.

### Agarose gel electrophoresis

All PCR products were electrophoresed on 2% agarose stained with ethidium bromide and visualized by UV transilluminator.

#### Semi-quantitative determination of PCR products

Semi- quantitation was performed using the gel documentation system (BioDO, Analyser) supplied by (Biometra) according to the following amplification procedure: Relative expression of each studied gene (R) was calculated following the formula:

R = Densitometrical units of each studied gene/ Densitometrical units of β-actin.

#### PCR detection of β-actin

Presence of RNA in all samples was assessed by analysis of the “house-keeping” gene β-actin. cDNA was generated from 1μg of total RNA extracted with AMV reverse transcriptase for 60min at 37°C. For PCR, 4 μl cDNA was incubated with 30.5 μl water, 4 μl 25 mM MgCl_2_, 1 μl dNTPs (10 mM), 5 μl ×10 PCR buffer, 0.5 μl (2.5U) Taq polymerase and 2.5 μl of each primer containing 10 pmol. β-actin primers (forward 5-TGTTGTCCCTGTATGCCTCT-3. reverse 5-TAATGTCACGCACGATTTCC-3) were designed from GenBank (accession no. J00691). The reaction mixture was subjected to 40 cycles of PCR amplification as follows: Denaturation at 95°C for 1min, annealing at 57°C for 1min and extension at 72°C for 2min. The PCR product yielded 206 bp fragments.

PCR amplification as follows: Denaturation at 95°C for 1min, annealing at 57°C for 1min and extension at 72°C for 2min. The PCR product yielded 206 bp fragments.

### Statistical analysis

Data were statistically described in terms of range, mean ± standard deviation (±SD), frequencies (number of cases) and relative frequencies (percentages) when appropriate. Comparison of quantitative variables between acanthosis nigricans (AN) and non (AN) groups was done using Mann– Whitney U test for independent samples. Comparison of quantitative variables between cases and control samples was done using Wilcoxon signed rank test. Correlations between various variables were made using the Spearman rank correlation equation. A probability value (*P* value) less than 0.05 was considered statistically significant. All statistical calculations were done using the computer programs: Microsoft Excel version 7 (Microsoft Corporation, NY, USA) and SPSS (Statistical Package for the Social Science; SPSS Inc., Chicago, IL, USA).

## Results

Mean skin tag number for participants was 40 ± 15.67. Skin tags were flesh-colored (F) in 7 participants and mixed (hyper pigmented and flesh color) (M) in 8 [Table 2] with no statistically significant difference between cases with acanthosis nigricans and those without. Seven patients exhibited acanthosis nigricans and eight did not. Mean body mass index (BMI) was 27.989 ± 4.5395. The internationally accepted interpretation for BMI is as follows: Underweight: < 18.5, normal: 18.5 to -24.9, overweight: 25.0 to - 29.9, obese: 30.0 to -39.9 and extremely obese: >40, which makes the participants mostly overweight.

**Table 2:** Color of skin tags

			Acanthosis negricans		Total
				
			No	Yes	
Color of skin tags	F*	Count	5	2	7
		%	62.5	28.6	46.7
	M†	Count	3	5	8
		%	37.5	71.4	53.3
*P value*				0.315	

* F for flesh;

† M for mixed (hyper pigmented and flesh colored)

**Figure 1:**
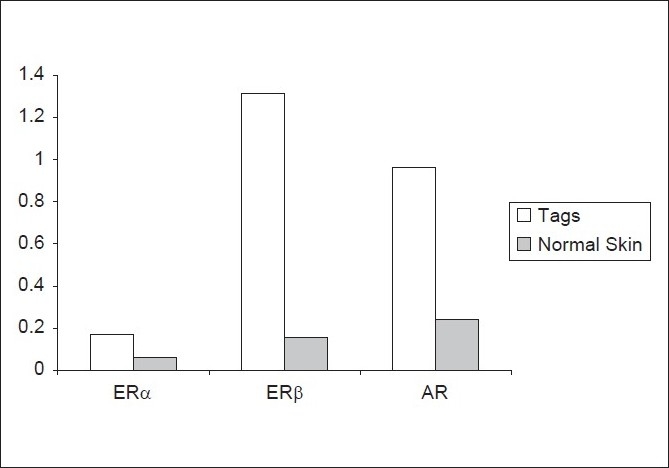
Mean receptor level in the skin tags versus normal skin

Average androgen receptors level (AR) was (0.9613 ± 0.61154) in skin tags and (0.2386 ± 0.23613) in control samples of healthy skin. This higher level in skin tags was statistically significant with a *P* value of 0.001 [Figure 1]. Estrogen receptors α level (ERα) was higher in skin tags (0.1693 ± 0.14714) than in control (0.0600 ± 0.03094). This difference was statistically significant, with a *P* value of 0.004 [Figure 1]. Similarly, estrogen receptors β level (ERβ) was higher in skin tags than it was in healthy skin, (1.3133 ± 0.56001) and (0.1573 ± 0.10754) respectively, a statistically significant finding (*P* value 0.001) [Figure 1].

**Figure 2:**
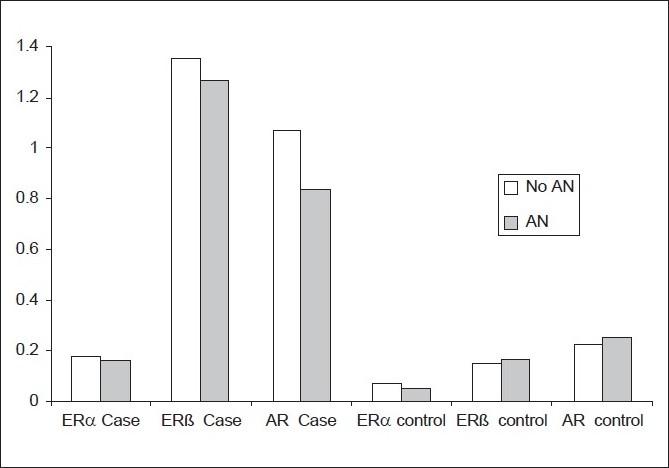
Mean receptor level in cases with Acanthosis nigricans (AN) versus those without (No AN)

No statistically significant difference was noted in receptor levels between participants with acanthosis nigricans and those without [Figure 2]. There was no significant correlation between body mass index and levels of androgen and estrogen receptor α and β [Table 3].

**Table 3:** Correlation of body mass index with androgen, estrogen receptor α and β levels

Correlations			BMI
Spearman's rho	ERα case	Correlation coefficient	−0.067
		Sig. (2-tailed)	0.813
		N	15
	ERβ	case Correlation coefficient	−0.164
		Sig. (2-tailed)	0.560
		N	15
	AR case	Correlation coefficient	−0.319
		Sig. (2-tailed)	0.246
		N	15
	ERα control	Correlation coefficient	0.311
		Sig. (2-tailed)	0.259
		N	15
	ERβ control	Correlation coefficient	0.387
		Sig. (2-tailed)	0.154
		N	15
	AR control	Correlation coefficient	−0.342
		Sig. (2-tailed)	0.212
		N	15

## Discussion

**Figure 3:**
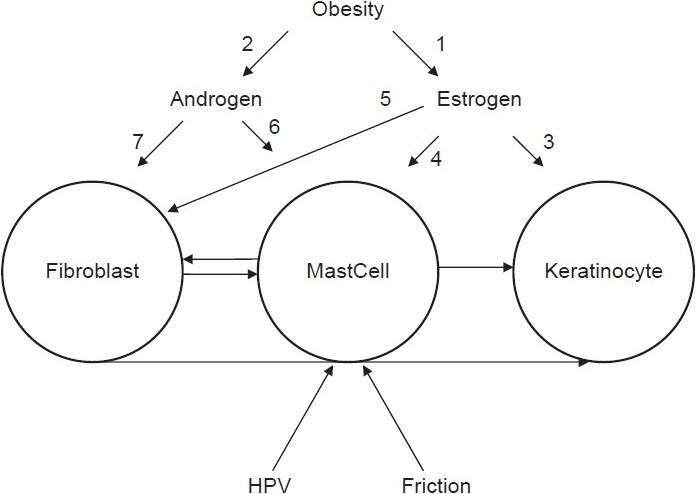
Suggested scenario of skin tags formation

Skin tags are related to obesity,[9] and a hormonal mechanism has been suggested in obese females, especially those with upper body obesity, where there is increased peripheral aromatization of androgens to estrogens[10] [See arrow-1 of Figure 3]. Similar hormonal imbalance exists in obese males where estrogen levels in blood increase in a direct proportion with body mass index (BMI).[11] Obese men have elevated levels of estrone, both free and total, as well as estradiol. Plasma estrogen levels do not decrease even after massive weight reduction.[12] This may be an underlying mechanism of skin tag development in obese men. However, our results contradict the fore mentioned study, where there was no statistically significant correlation between BMI and androgen or estrogen receptors levels as our participants were overweight non diabetic.

Our results point out to a significant estrogen receptor levels and the development of skin tags, which in turn explains the absence of skin tags before puberty and the arrest of their development following menopause.

The following is a possible cascade of events that explains the role of estrogen concentration and receptor levels in the development of skin tags on the cellular scale: Keratinocytes express both estrogen receptors (ERα and ERβ) [See arrow-3 of Figure 3]. Estradiole binds to keratinocytes with high affinity, and in its normal physiological range increases the regulation of the level of ERβ receptors and induces keratinocyte proliferation.[13] In addition, estrogen targets human skin fibroblasts. 17β-estradiol has been shown to increase fibroblast proliferation in human skin[14] [See arrow-5 of Figure 3]. Finally, there is a role for mast cells interacting with fibroblasts and keratinocytes in the course of the development of skin tags [arrows without number in Figure 3].[15] Mast cells express high-affinity estrogen receptors [See arrow-4 of Figure 3]. 17 β-estradiol augments secretion of histamine and serotonin.[16] Binding of estradiol, in physiological concentrations, to a membrane estrogen receptor α, initiates a rapid onset, progressive influx of extra-cellular Ca(2+), inducing exocytosis of mast cell products.[17]

A similar role can be hypothesized for androgens and their receptors, especially in obese females with upper body obesity who exhibit insulin resistance, hyperandrogenemia, hyperinsulinemia, altered gonadotrophin and insulin-like growth factor binding proteins (IGFBPs), increased leptin levels and altered neuro-regulation of hypothalamic-pituitary-gonadal axis[10] [See arrow-2 of Figure 3]. This role is supported by our results and by previous observations. It was found that blocking androgen receptors with sex hormone antagonists in the testis of frog *Rana esculenta* causes a significant increase in mast cell count, supporting the involvement of androgens in mast cell proliferation and/or differentiation[16] [See arrow-6 of Figure 3]. On the contrary, it was found that testosterone and tamoxifen have an inhibitory effect on mast cell secretion of histamine and serotonin in rats.[18] Considering the fact that mast cells are a major player in the development of skin tags,[15] and the effect of androgens on their concentration and behavior, androgens can have a role in the pathogenesis of skin tags. Despite the reported absence of androgen receptors in the epidermis,[19] dermal fibroblasts in androgen dependent areas have androgen receptors with binding affinity for circulating androgens[20] [See arrow-7 of Figure 3].

According to our results, the higher level of estrogen α and β as well as the androgen receptors in association with skin tags, represents strong evidence of the pathogenesis of sex hormones in this disease, and propose that the neck is an androgen dependent area just similar to the axillae and the groins, though hairless.
